# Anticancer activity of bergenin against cervical cancer cells involves apoptosis, cell cycle arrest, inhibition of cell migration and the STAT3 signalling pathway

**DOI:** 10.3892/etm.2021.10085

**Published:** 2021-04-19

**Authors:** Xingyuan Shi, Manman Xu, Kai Luo, Wenjin Huang, Hongwei Yu, Tongchong Zhou

Exp Ther Med 17: 3525-3529, 2019; DOI: 10.3892/etm.2019.7380

Following the publication of the above paper, a concerned reader drew to the Editor’s attention that, in Fig. 6, the data panels shown for the experiments performed with 7.5, 15 and 30 μM bergenin were strikingly similar in terms of their overall appearance. Furthermore, several figures contained data that bore striking similarities to data published in other papers; notably, the cellular data shown in Fig. 3 appeared to have been presented with a different field of view in Fig. 2 of another paper written by different authors and based at a different research institution [Zeng C, Guo B, Chen J and He W: Antitumor effects of chrysophanol in malignant optic nerve meningioma cell lines are mediated via caspase activation, induction of mitochondrial mediated apoptosis, mitochondrial membrane depolarization and targeting the mitogen-activated protein kinase signaling pathway. Pharmacology 104: 28-35, 2019].

After having conducted an independent investigation in the Editorial Office, the Editor of *Experimental and Therapeutic Medicine* has determined that this article should be retracted from the Journal on account of a lack of confidence concerning the originality and the authenticity of the data. The authors were asked for an explanation to account for these concerns, but the Editorial Office never received any reply. The Editor regrets any inconvenience that has been caused to the readership of the Journal.

